# Establishment of a qualified integrated care system after total knee arthroplasty as a role of regional rheumatoid and degenerative arthritis centers

**DOI:** 10.1186/s12877-022-03277-z

**Published:** 2022-07-21

**Authors:** Eun Young Han, Sang Rim Kim, Kye Hee Cho, Sang Hee Im

**Affiliations:** 1 Department of Rehabilitation Medicine, Regional Rheumatoid and Degenerative Arthritis Center, Jeju National University College of Medicine, Jeju National University Hospital, Jeju, Republic of Korea; 2 Department of Orthopedic Surgery, Regional Rheumatoid and Degenerative Arthritis Center, Jeju National University College of Medicine, Jeju National University Hospital, Jeju, Republic of Korea; 3 Department of Rehabilitation Medicine, CHA Ilsan Medical Center, CHA University, Goyang, Republic of Korea; 4 Department and Research Institute of Rehabilitation Medicine, Severance Hospital, Yonsei University College of Medicine, Seoul, Republic of Korea

**Keywords:** Arthroplasty, Knee, Osteoarthritis, Rehabilitation, Aged

## Abstract

**Background:**

The geriatric population and advanced knee osteoarthritis are rapidly increasing in Korea, and the socioeconomic burden of total knee arthroplasty (TKA) is increasing. This study aimed to analyze the demographic, clinical and socioeconomic characteristics of patients who underwent TKA and to differentiate the factors affecting participation in inpatient-intensive rehabilitation programs after TKA in the Jeju regional rheumatoid and degenerative arthritis center established by the government.

**Methods:**

This retrospective cohort study included 845 patients (735 females; 72.0 ± 5.8 years) diagnosed with primary osteoarthritis (OA) of the knee who underwent elective unilateral primary TKA between January 2013 and June 2016. Demographic, clinical, and socioeconomic characteristics, including age, body mass index, obesity, length of stay, OA severity, underlying disease, education level, occupation, and location of residence were reviewed. Patients were allocated to the TKA-only group (home discharge) and to the TKA + rehab group (participation in post-TKA rehabilitation). The variables were analyzed and compared before and after the establishment of the center and according to participation in intensive rehabilitation.

**Results:**

Patients who underwent TKA were mostly female, in the 60 s, and had a high prevalence of comorbidities and obesity. After the rehabilitation center's establishment, the intensive post-TKA participation increased profoundly from 3% to 59.2%. Participants after the center establishment had lower mean BMI and a higher proportion of K-L grade 4 compared to those before the center establishment. The location of residence was the only factor differentiating the participation in the intensive rehabilitation.

**Conclusion:**

The regional rheumatoid and degenerative arthritis center was appropriate to satisfy the high unmet need for participating in the intensive rehabilitation after TKA and to execute the qualified integrated post-TKA care system. Policy support should ensure the early rehabilitation and a qualified integrated care system and prepare for the increased burden of revision. Future longitudinal studies should be conducted to assess the long-term effect of the integrated post-TKA rehabilitation program on functional outcomes and patient survivorship free from revision.

## Background

The geriatric population is rapidly growing in South Korea, and osteoarthritis (OA) is one of the most common degenerative bone and joint diseases in geriatrics, and the knee is most involved. Patients with knee OA complain of severe pain that causes gait disturbance and frailty [1]. Total knee arthroplasty (TKA) is an effective method for treating advanced knee arthritis [2], and primary and revision TKAs have tremendously increased from 2001 to 2010 by 407% and 267%, respectively [3]. The socioeconomic burden of TKA is also increased in Korea, and TKA is the 6^th^ most common surgical procedure in Korea. In terms of overall cost, it is the fourth highest, and the days of hospitalization per surgery case was longest in 2018 [4]. Although the National Health Insurance (NHI) system and Health Insurance Review and Assessment Service (HIRA) provide universal coverage in Korea, most patients should pay out-of-pocket (OOP) for health care, which accounts for approximately 32–26% of annual total household health care spending [5] and may cause an inequality in accessing medical services among variable socioeconomic groups, and further accelerate the overconcentration of medical resources and health care utilization of patients in metropolitan areas. Thus, the Ministry of Health and Welfare established and has been operating five regional rheumatoid and degenerative arthritis centers to promote decentralization of medical resource utilization and to improve the quality of medical services via a multidisciplinary care approach among orthopaedic surgeons, rheumatologists, and physiatrists. Three centers (Chungnam, Chonnam and Jeju) opened in 2013, one (Dae gu Catholic) in 2014 and one (Gyeongsang) in 2016.

The Jeju regional rheumatoid and degenerative arthritis center opened on 25 September 2013 and applied a critical pathway incorporating intensive rehabilitation therapy after TKA. As the coverage of NHIs and HIRAs focused on TKA only in Korea, studies are very limited for post-TKA rehabilitation therapy.

Therefore, the objectives of this study were to analyze the demographic, clinical and socioeconomic characteristics of patients who underwent TKA and to differentiate the factors affecting the participation in the inpatient-intensive rehabilitation programs after TKA as prerequisites to establish a qualified integrated care system after TKA as a role of regional rheumatoid and degenerative arthritis centers.

## Methods

### Participants and medical team

This retrospective cohort study included 845 patients (735 females and 70 males; average age, 72.0 ± 5.8 years) diagnosed with primary OA of the knee who underwent elective unilateral primary TKA at the Department of Orthopaedic Surgery in Jeju National University Hospital between January 2013 and June 2016. Home discharged patients were allocated to the TKA-only group, and patients discharged or transferred to the Department of Rehabilitation for intensive rehabilitation programs were assigned to the TKA + rehab group.

Team members of the qualified Integrated Care System consisted of one orthopaedic surgeon, 3 physiatrists, 2 nurses (orthopaedic and rehabilitation coordinators), and 4 physical therapists. The inclusion criteria consisted of the ability to walk independently with or without an ambulatory aid. The exclusion criteria were as follows: 1) previous neurological or orthopaedic disease disturbing ambulation, such as orthopaedic injury, previous joint surgery in the lower extremities, or unstable cardiorespiratory disease; 2) bilateral TKA; 3) revision TKA; 4) TKA surgery at another hospital; and 5) combined severe medical complications.

### Demographic and clinical data

Demographic characteristics included age, sex, weight, height, and body mass index (BMI) which was calculated as the weight (kg) divided by the height (m^2^). Individuals aged 65 years or older and 80 years or older were classified as the elderly and very elderly, respectively, and we also classified those aged 70 years or over as the old-elderly. The obesity was defined by the modified Asia–Pacific criteria (BMI ≥ 25.0 kg/m^2^) [6]. The presence of documented comorbidities such as hypertension (HTN) and diabetes mellitus (DM) was also collected.

Patients were asked to mark the level of knee pain using a visual analogue scale (VAS) preoperatively. The scale consisted of a 10 cm-long horizontal line ranging from 0 (worst, totally unsatisfied) to 10 (best, completely satisfied) points with respective facial expressions above the line [7]. The severity of knee osteoarthritis was classified by Kellgrene-Lawrence (K-L) grade using anterior posterior (AP) radiographs as follows [8]: 1) grade 1, doubtful narrowing of the joint space with possible osteophyte formation; 2) grade 2, possible narrowing of the joint space with definite osteophyte formation;3) grade 3, definite narrowing of joint space, moderate osteophyte formation, some sclerosis, and possible deformity of bony ends; and 4) grade 4, large osteophyte formation, severe narrowing of the joint space with marked sclerosis, and definite deformity of bone ends.

The intensive rehabilitation program consisted of progressive resistance exercises (including leg press, leg extension, leg curl, hip abduction, and hip adduction) using pneumatic resistance machines (HUR SmartTouch®, HUR Co., Kokkola, Finland) and progressive gait training using a lower-body positive pressure treadmill (G trainer®, Alter-G Inc., Fremont, CA, USA), and aerobic exercise using a stationary ergometer [9]. A skilled physical therapist evaluated 1RM for each equipment and set the appropriate resistance exercise intensity and saved the personalized training program for HUR SmartTouch system. The machine automatically loads personalized training program for each patient with adjusted seat position and resistance settings. The intensity of resistance exercise advanced gradually from 30% (3 sets of 15 repetitions) to 60% of 1RM; the number of repetitions decreased as the load increased. One physiotherapist supervised up to 4 patients.

For progressive gait training, a lower-body positive pressure treadmill was used under the supervision of a physical therapist. Initially, bodyweight support (BWS) and speed were set to 50% and 2.0 km/hr; BWS was gradually reduced, while speed increased. The G-trainer was used daily, whereas the ergometer and HUR machine were used every other day. The TKA rehabilitation programs were performed as follows: 30 min per session, 2 sessions per day, 5 days per week, for 2 weeks. The parameters were also compared before and after the establishment of the center (BEC and AEC).

### Socioeconomic characteristic

**Fig. 1:**
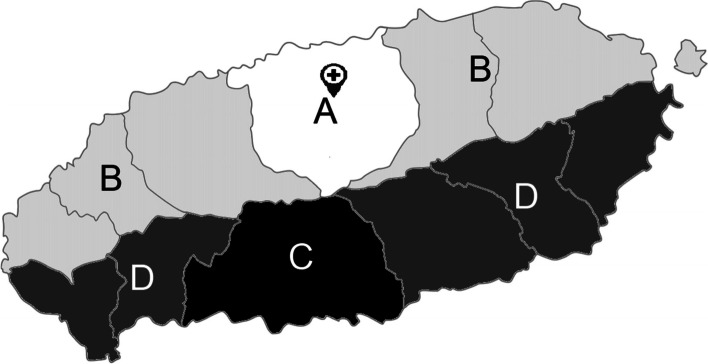
Current and previous administrative divisions of Jeju Island. The cross indicates the location of Jeju regional rheumatoid and degenerative arthritis center. The previous and current administrative districts are as indicated below. A: previous Jeju city district  B: previous North Jeju County district  C: previous Seogwipo City district  D: previous South Jeju County district  A and  B: current Jeju city district  C and  D: current Seogwipo city district

The locations of residence were classified using the previous addresses (Jeju City, Seogwipo City, North Jeju County, South Jeju County) and current administrative districts (Jeju and Seogwipo), which had been reorganized since 2006. We also investigated whether they lived in urban areas, including Jeju City and Seogwipo City, and whether they lived in Jeju City where the Jeju regional rheumatoid and degenerative arthritis center was (Fig. 1).

The date of admission, date of discharge, length of hospital stay (LOS), in-hospital complications, medical insurance status (National Health Insurance; NHI, Medicaid) and education level were also retrieved.

The level of education was categorized depending on the highest diploma that the patient acquired as follows: no formal education, primary education (elementary diploma), secondary education (middle or high school or 2-year college diploma), and finally higher education (university or graduate school diploma) [10].

### The classification of occupation

We also evaluated the job that the patient had for the longest duration according to the Korean Standard Classification of Occupations [11]. These occupations were categorized into three groups depending on the degree of physical work: nonmanual workers (managers, professionals and related workers, and clerks), service or sales workers, and manual workers (skilled agricultural, forestry, fishery, craft, and related trades workers, equipment, machine operating and assembling workers, and elementary workers) [12].

### Statistical analysis

For statistical analysis, standard statistical software (SPSS version 20.0 for Windows, SPSS, Inc., Chicago, IL, USA) was used. All descriptive statistics were used, and the data are presented as the mean ± standard deviation (SD). Nominal and categorical variables were analyzed by χ^2^ tests. Baseline demographic and clinical characteristics were compared between the TKA-only and TKA + rehabilitation groups and between the BEC and AEC groups using independent sample t-tests for continuous data. A *p*-value < 0.05 was considered significant.

## Results

A total of 166 patients were enrolled between January and September 2013, and 679 patients were enrolled from October 2013 to June 2016. The annual numbers of primary TKAs were 227, 225 and 258 in 2013, 2014 and 2015, respectively, and the total number of primary TKAs was 135 during the first 6 months in 2016. There were significant annual differences in the prevalence of obesity and proportions of manual workers and males, especially in 2015 (Table 1).

**Table 1:** Comparison of general, clinical and socioeconomic characteristics between 2013 and 2015

Variables	2013	2014	2015	*p* value
Numbers	227	225	258	
Age	71.7 ± 5.9	71.3 ± 6.2	72.7 ± 5.4	0.24
≤ 59	5 (2.2)	6 (2.7)	4 (1.6)	0.094
60–69	76 (33.5)	79 (35.1)	61 (23.3)	
70–79	126 (55.5)	125 (55.6)	170 (65.9)	
≥ 80	20 (8.8)	15 (6.7)	23 (8.9)	
Elderly (≥ 65)	204 (89.9)	200 (88.9)	239 (92.6)	0.339
Old-elderly (≥ 70)	146 (64.3)	141 (62.3)	193(74.8)	0.006 ^**^
Very elderly (≥ 80)	20 (8.8)	15 (6.7)	23 (8.9)	0.609
Sex (female: male)	206 (90.7): 21 (9.3)	201 (89.3): 24 (10.7)	212 (82.2): 46 (17.8)	0.009 ^**^
Op site (Right)	93 (41.0)	92 (40.9)	119 (46.1)	0.404
BMI	26.8 ± 3.2	26.1 ± 3.5	26.3 ± 3.6	0.076
Weight	62.2 ± 9.3	61.7 ± 9.4	62.7 ± 10.5	0.546
Obesity	165 (72.7)	130 (57.8)	154 (59.7)	0.002 ^**^
LOS	16.7 ± 2.2	16.8 ± 3.5	16.3 ± 1.5	0.08
VAS	5.7 ± 0.7	5.8 ± 0.4	5.8 ± 0.5	0.052
K-L grade				
3/4	75 (33.0)/152 (67.0)	59 (26.2)/166 (73.8)	63 (24.4)/195 (75.6)	0.088
Education				
No formal education	31 (13.7)	28 (12.4)	12 (4.7)	0.001 ^**^
Occupation				
Manual work	16 (7.0)	34 (15.1)	133 (51.6)	< 0.001 ^**^
Urban residence				
Urban: Country	137 (60.4): 90 (39.6)	132 (58.7): 93 (41.3)	152 (58.9): 106 (41.1)	0.924
Location of residence (Current administrative districts)				
Jeju: Seogwipo	149 (65.6): 78 (34.4)	148 (65.8): 77 (34.2)	174 (67.4): 84 (32.6)	0.895
Former administrative districts				
Jeju city	111 (48.9)	104 (46.2)	122 (47.3)	0.848
Medical insurance				
NHI: Medicaid	209 (92.1): 18 (7.9)	215 (95.6): 10 (4.4)	249 (96.5): 9 (3.5)	0.074

Values are mean ± standard deviation or numbers (%)

*Op* operation, *BMI* body mass index, *LOS* length of stay, *VAS* visual analogue scale, *K-L* Kellgrene-Lawrence, *NHI* national health insurance

Only 3% of the patients were transferred or admitted to the Department of Rehabilitation in the BEC group, whereas 59.2% agreed with intensive post-TKA rehabilitation in the AEC group. The BEC group showed a higher mean BMI and prevalence of obesity but a lower proportion of patients with K-L grade 4 (27.1 ± 3.2 kg/m^2^, 65.7%) than the AEC group (26.2 ± 3.5 kg/m^2^, 75.7%) (Table 2). The distributions of education level were significantly different between BEC and AEC groups and the proportion of the patients who did not have any formal education was higher, and the proportion of the manual workers were fewer in BEC group (14.5%, 7.2%) than in AEC group (7.1%, 33.3%), but there were no significant differences between BEC and AEC groups in the residence-related factors (Table 3). After EC, 59.2% participated in the intensive post-TKA rehabilitation. The BEC group had significantly higher proportions of patients who lived in Jeju of both former and current administrative districts than the AEC group (Tables 3).

**Table 2:** General and clinical characteristics in all patients and comparison before and after the establishment of regional rheumatoid and degenerative arthritis center

Variables	All	BEC	AEC	*p* value
Numbers	845	166 (19.6)	679 (80.4)	
Age	72.0 ± 5.8	71.1 ± 5.8	72.2 ± 5.7	0.03 ^*^
≤ 59	16 (1.9)	4 (2.4)	12 (1.8)	0.108
60–69	258 (30.5)	63 (38.0)	195 (28.7)	
70–79	501 (69.3)	88 (53.0)	413 (60.8)	
≥ 80	70 (8.3)	11 (6.6)	59 (8.7)	
Elderly (≥ 65)	769 (91.0)	147 (88.6)	622 (91.6)	0.227
Old-elderly (≥ 70)	571 (67.6)	99 (59.6)	472 (69.5)	0.016 ^*^
Very elderly (≥ 80)	70 (8.3)	59 (8.7)	11 (6.6)	0.436
Sex (female)	735 (87.0)	151 (91.0)	584 (86.0)	0.095
Op site (Right)	361 (42.7)	73 (44.0)	288 (42.4)	0.727
BMI	26.4 ± 3.4	27.1 ± 3.2	26.2 ± 3.5	0.003 ^**^
Weight	62.1 ± 9.7	62.8 ± 8.9	62.0 ± 9.9	0.323
Obesity	528 (62.5)	124 (74.7)	404 (59.5)	< 0.001 ^**^
LOS	16.6 ± 2.4	16.7 ± 2.3	16.5 ± 2.5	0.371
Rehabilitation	407 (48.2)	5 (3.0)	402 (59.2)	< 0.001 ^**^
VAS	5.8 ± 0.5	5.7 ± 0.8	5.8 ± 0.4	0.094
K-L grade				
3/4	222 (26.3)/623 (73.7)	57 (34.3)/109 (65.7)	165 (24.3)/514 (75.7)	0.01 ^*^
Underlying diseases				
HTN	564 (66.7)	121(72.9)	443 (65.2)	0.066
DM	174 (20.6)	34 (20.5)	140 (20.6)	1

Values are the mean ± standard deviation or numbers (%)

*EC*, the establishment of regional rheumatoid and degenerative arthritis center, *BEC* before EC, *AEC* after EC, *Op* operation, *BMI* body mass index, *LOS* length of stay, *VAS* visual analogue scale, *K-L* Kellgrene-Lawrence, *HTN* hypertension, *DM* diabetes mellitus

^*^ *p* < 0.05

^**^ *p* < 0.01

**Table 3:** Socioeconomic characteristics of all patients and comparisons before and after the establishment of the regional rheumatoid and degenerative arthritis center

Variables	All	BEC	AEC	*p* value
Education				0.001 ^**^
No formal education	72 (8.5)	24 (14.5)	48 (7.1)	0.005 ^**^
Primary education	260 (30.8)	62 (37.3)	198 (29.2)	
Secondary education	490 (58.0)	76 (45.8)	414 (61.0)	
Higher education	23 (2.7)	4 (2.4)	19 (2.8)	
Occupation				
Manual work	238 (28.2)	12 (7.2)	226 (33.3)	< 0.001 ^**^
Urban residence				
Urban: Country	509 (60.2): 336 (39.8)	103(62.0): 63 (38.0)	406 (59.8): 273 (40.2)	0.658
Location of residence (Current administrative districts)				
Jeju: Seoqwipo	557 (65.9): 288 (34.1)	105 (63.3): 61 (36.7)	251 (57.3): 187 (42.7)	0.465
Former administrative districts				
Jeju city	402 (47.6)	80 (48.2)	322 (47.4)	0.863
Medical insurance				
NHI: Medicaid	799 (94.6): 46 (5.4)	152 (91.6): 14 (8.4)	647 (95.3): 32 (4.7)	0.083

Values are the mean ± standard deviation or numbers (%)

*EC*, the establishment of regional rheumatoid and degenerative arthritis center, *BEC* before EC, *AEC* after EC, *NHI* national health insurance

^*^ *p* < 0.05

^**^ *p* < 0.01

There was no significant different between rehabilitation and home discharge groups in general and clinical characteristics (Table 4) and the proportion of Jeju city residence in both former and current administrative districts was significantly high, and the education level was significantly different between two groups. (Table 5).

**Table 4:** General and clinical characteristics of patients after the establishment of regional rheumatoid and degenerative arthritis centers and comparisons between the home discharge and rehabilitation groups

Variables	TKA + Rehab	TKA only	*p* value
Numbers	402 (59.2)	277 (40.8)	
Age	72.1 ± 5.9	72.2 ± 5.6	0.33
≤ 59	7 (1.7)	4 (2.5)	0.436
60–69	121 (30.1)	62 (38.5)	
70–79	235 (58.5)	85 (52.8)	
≥ 80	39 (9.7)	10 (6.2)	
Elderly (≥ 65)	366 (91.0)	256 (92.4)	0.575
Old-elderly (≥ 70)	278 (68.3)	293 (66.9)	0.713
Very elderly (≥ 80)	39 (9.7)	20 (7.2)	0.271
Sex	343 (85.3)	241 (87.0)	0.575
Op site (Right)	173 (43.0)	115 (41.5)	0.752
BMI	26.0 ± 3.4	26.4 ± 3.5	0.204
Weight	61.9 ± 9.7	62.4 ± 9.8	0.441
Obesity	227 (56.5)	177 (63.9)	0.601
LOS	16.5 ± 2.7	16.5 ± 2.1	0.908
VAS	5.8 ± 0.4	5.8 ± 0.5	0.294
K-L grade			
3/4	110 (27.0)/297 (73.0)	112 (25.6)/326 (74.4)	0.64
Underlying diseases			
HTN	266 (66.2)	177 (63.9)	0.566
DM	82 (20.4)	28 (20.9)	1

Values are the mean ± standard deviation or numbers (%)

*Op* operation, *BMI* body mass index, *LOS* length of stay, *VAS* visual analogue scale, *K-L* Kellgrene-Lawrence, *HTN* hypertension, *DM* diabetes mellitus

^*^ *p* < 0.05

^**^ *p* < 0.01

**Table 5:** Socioeconomic characteristics of patients after the establishment of regional rheumatoid and degenerative arthritis centers and comparisons between the home discharge and rehabilitation groups

Variables	TKA + Rehab	TKA only	*p* value
Education level			0.034 ^*^
No formal education	26 (6.5)	22 (7.9)	0.543
Primary education	133 (33.1)	65 (23.5)	0.035 ^*^
Secondary education	230 (57.2)	184 (66.4)	0.035 ^*^
Higher education	13 (3.2)	6 (2.2)	0.483
Occupation			
Manual work	140 (34.8)	86 (31.0)	0.321
Urban residence (Jeju city and Seogwipo city)			
Urban: Country	242 (60.2): 160 (39.8)	164 (59.2): 113 (40.8)	1
Location of residence (Current administrative districts)			
Jeju: Seogwipo	303 (75.4): 99 (24.6)	149 (53.8): 128 (46.2)	< 0.001 ^**^
Formal administrative districts			
Jeju city	213 (53.0)	109 (39.4)	0.001 ^**^
Medical insurance			
NHI: Medicaid	385 (94.6): 22 (5.4)	414 (94.5): 24 (5.5)	1

Values are the mean ± standard deviation or numbers (%)

*NHI* national health insurance

^*^ *p* < 0.05

^**^ *p* < 0.01

## Discussion

This study confirmed the demographic, clinical and socioeconomic characteristics of patients who underwent TKA. After the EC, the need for an inpatient-intensive rehabilitation program after TKA increased from 3% to 59.2% in the Jeju regional rheumatoid and degenerative arthritis center.

It also indicated that the total number of TKAs had been steady or had increased gradually after EC by 114% between 2014 and 2015. A recent large-scale study based on HIRA registries reported TKA procedure rates of 155.5 (2013), 155.8 (2014) and 173.3 (2015) per 100,000 people [3]. There was a gradual increase in TKA growth in Korea from 2010 to 2018, despite the dramatic increase in the growth rate before 2010 [3]. This may have been caused by improved accessibility to the medical system as well as an increase in the geriatric population [21]. They showed an increasing tendency of the old-elderly from 48.1% (2010) to 61.0% (2018) and higher proportions of females and geriatrics and a substantial prevalence of comorbidities such as HTN and DM [3]. In parallel with this result, our data indicated that approximately 90% of all participants was the elderly (age ≥ 65); the mean age (72.0 ± 5.8 years) was in the early 70 s, the optimal age for TKA that could achieve the maximal passive range of motion without increasing the rate of revision or mortality [13].

Interestingly, the number of males, geriatrics, and manual workers increased. The proportion of male sex and old-elderly has significantly changed from 9.3% and 64.3% (2013) to 10.7% and 62.3% (2014), and 17.8% and 74.8% (2015) (Table 1). The life expectancy was shorter in males about 6 years, according to 2015 Population Census report of Republic of Korea [14]. Weighing the possible risk and benefits of TKA may have resulted in female predominance. However, as the male life expectancy increased from 76.84 years (2010) to 78.96 years (2015) and 79.70 years (2018) and the westernization of lifestyle prevented rapid osteoarthritis progression, the male candidates for TKA may also have increased.

On the contrary, the demographic composition of this study showed a slightly different pattern than the general data on residence and occupation [14]. Our results indicated that a total of 65.9% lived in Jeju city of current administrative districts and the manual workers accounted for 28.2% of all participants, approximately 20% of Jeju and 33% of Seogwipo. Considering the overall population distribution of Jeju province in which 73.4% resides in Jeju city and 26.6% in Seogwipo city [14], the higher proportion of Seogwipo residence in this study may have contributed to the greater proportion of the elderly and the agricultural workers. Data of Korean National Statistics demonstrated high proportion of elderly and agricultural households in Seogwipo city [15].

On the other hand, the mean duration of LOS in our study was 16.7 ± 2.2 days. Although it may be longer than those of Western countries such as the United States (3.6 ± 1.7 days) [16] and Denmark (3 ± 3 days in elective cases, 5 ± 6 days in nonelective cases) [17], it was notably shorter than the average LOS on HIRA (21.2 days in 2018). The mean LOS in Western countries may be much shorter because it may not be counted as LOS if TKA patients visit outpatient rehabilitation clinic or discharged to a rehabilitation facility.

Generally, the national medical insurance systems guarantee coverage to treatment for a longer LOS only for indicated reasons such as bilateral arthroplasty, intractable pain, gait instability, and other perioperative complications [18]. However, the demographic and clinical characteristics of TKA in Korea are quite different from most of the developed Western countries; the revision rates are much low, despite the similar rate of primary TKA [21]. On the other hand, the mean duration of LOS in this study was relatively consistent regardless of EC or rehabilitation. This might be explained by the fact that the hospital preferred readmission after discharge rather than transfer to the inpatient rehabilitation ward because the Korean HIRA cut or reduced the Medicare payment if the LOS was more than 15 days. Interestingly, the national medical insurance systems of Japan supported coverage for much longer LOS (35.1 ± 1.7 days) than Korea [19] and also provided comprehensive rehabilitation therapy in long-term care wards for community-based integrated care systems [20].

As the aging of the Korean population is unprecedentedly rapid, the policymakers and healthcare providers should prepare for comorbidity care and revision burdens in the elderly and the very elderly. A previous study suggested further study based on a comprehensive analysis of various patients’ demographical and clinical information [21]. Thus, the regional rheumatoid and degenerative arthritis center is an emerging experimental model for managing arthritis, comorbidities, and perioperative side effects, and integrating medical and surgical treatment with rehabilitation in Korea. Considering the average LOS (21 days) and lack of long-term care wards for TKA in Korea, policy support would be necessary to ensure sufficient days of hospitalization after TKA and build incentive programs for early rehabilitation and a qualified integrated care system.

This study was the first to review of various characteristics of patients undergoing TKA in the regional rheumatoid and degenerative arthritis center, and it was comparable to representing TKA in the Korean general population. Notably, the need for post-TKA rehabilitation was very high, but the main factor differentiating participation in rehabilitation was the location of residence despite slightly different distributions of education levels between groups (Table 5). Thus, establishing a regional rheumatoid and degenerative arthritis center in the respective residency is appropriate for the integrated care of arthritis in geriatrics.

This study had some limitations. First, it was a retrospective cohort study, and the available demographical and clinical data were very limited, especially in the TKA-only group. Second, it was a cross-sectional analysis without follow-up. Thus, we could not analyze the effect of preoperative status or monitor functional changes between admission and discharge. Third, we excluded revision procedures and could compare primary TKA with revision. Third, the practice patterns may differ depending on each facility and region, which may have affected LOS, and the participation in rehabilitation. Finally, this study has a limitation in exploring the causative and longitudinal relationship of rehabilitation therapy with functional outcome.

In conclusion, patients who underwent primary TKA in the Jeju regional rheumatoid and degenerative arthritis center were mostly female, aged in the 60 s, and had a high prevalence of comorbidities and obesity. Additionally, the only factor differentiating participation in intensive rehabilitation was the location of residence. Thus, the regional rheumatoid and degenerative arthritis center was appropriate to meet the high need for participating in intensive rehabilitation after TKA and for the qualified integrated post-TKA care system. Furthermore, policy support should ensure adequate hospital stay after TKA, build incentive programs for early rehabilitation and qualified integrated care systems and prepare for the increased burden of revision, and future longitudinal studies should be conducted to assess the long-term effect of the integrated post-TKA rehabilitation program on functional outcomes and patient survivorship free from revision.

## Data Availability

The datasets used and/or analyzed during the current study are available from the first and corresponding authors on reasonable request.

## Abbreviations

AEC: After the establishment of regional rheumatoid and degenerative arthritis center
AP: Anterior posterior
BEC: Before the establishment of regional rheumatoid and degenerative arthritis center
BMI: Body mass index
DM: Diabetes mellitus
HIRA: Health Insurance Review and Assessment Service
HTN: Hypertension
K-L: Kellgrene-Lawrence
LOS: Length of hospital stay
NHI: National Health Insurance
OA: Osteoarthritis
OOP: Pay out-of-pocket
Op: Operation
TKA: Total knee arthroplasty
VAS: Visual analogue scale
